# Perceptions, attitudes, and knowledge of teachers serving as mental health lay counselors in a low and middle income country: a mixed methods pragmatic pilot study

**DOI:** 10.1186/s13033-021-00453-3

**Published:** 2021-04-29

**Authors:** Christina M. Cruz, Molly M. Lamb, Priscilla Giri, Juliana Vanderburg, Peter Ferrarone, Surekha Bhattarai, Aileen A. Giardina, Karen Hampanda, Bradley N. Gaynes, Michael Matergia

**Affiliations:** 1 Department of Psychiatry, University of North Carolina at Chapel Hill School of Medicine, 101 Manning Drive, CB #7160, Chapel Hill, NC 27599 USA; 2 Department of Epidemiology, Colorado School of Public Health at the University of Colorado Anschutz Medical Campus, 13001 E. 17th Place, Mail Stop C245, Aurora, 80045 CO USA; 3 Center for Global Health, Colorado School of Public Health at the University of Colorado Anschutz Medical Campus, 131999 E. Montview Blvd., Suite 310, Mail Box A090, Aurora, CO 80045 USA; 4 Darjeeling Ladenla Road Prerna, 42 Ladenla Road, Darjeeling, West Bengal 734101 India; 5 School Psychology Program, University of North Carolina at Chapel Hill School of Education, 100 E. Cameron Ave, Chapel Hill, NC 27514 USA; 6 Department of Global Health and Development, London School of Hygiene and Tropical Medicine, London, UK; 7 Broadleaf Health and Education Alliance, 919 Main Street, Stroudsburg, PA 18360 USA; 8 Department of Obstetrics and Gynecology, University of Colorado Anschutz Medical Campus, Aurora, CO USA

**Keywords:** Teacher, Lay counselor, Task-shifting, Child mental health, School mental health, Perceptions, Attitude, Knowledge, LMIC

## Abstract

**Background:**

Low and middle-income countries (LMICs) lack trained child mental health professionals. While teachers’ child development experience potentially positions them to fill the gap as lay mental health counselors, they have rarely delivered indicated child mental health care in LMICs. As part of assessing the feasibility of teachers serving as lay counselors, we explored teachers’ perceptions of serving as lay counselors and their mental health attitudes and knowledge.

**Methods:**

In 2018, with training and supervision, 19 primary school teachers from five rural, low cost private schools in Darjeeling, India, served as lay counselors in their classrooms. Using mixed methods, we examined teacher perceptions of serving as lay counselor and mental health attitudes and knowledge through a survey (n = 15), a summative assessment (n = 14), and semi-structured interviews (n = 17). For the survey and summative assessment, pre-training, post-training, and post-intervention mean scores were compared using paired *t* tests. Post-intervention interviews were coded for teachers’ perceptions of serving as lay counselor and mental health attitudes and knowledge.

**Results:**

Qualitatively, teachers expressed being willing to serve as lay counselor, having more inclusive mental health attitudes, and retaining mental health knowledge as applicable to use during instructional time or incorporation into the knowledge transfer process, their primary duty. By contrast, quantitatively, teachers’ attitudes appeared to become more inclusive on the study-specific survey pre versus post-training, but reverted to pre-training levels post-intervention. Teachers’ mental health knowledge on the summative assessment did not change pre-training versus post-training versus post-intervention.

**Conclusions:**

Training, supervision, and serving as lay counselors led to teachers’ willingness to serve as lay counselors. Teachers served as lay counselors by utilizing therapeutic techniques during class time and incorporating them into their typical instruction, not through delivering traditional office-like care. Teacher practices may be pointing to the potential emergence of an “education as mental health therapy” system of care. Their changes in attitudes and knowledge reflected their emerging practices. Quantitative measures of knowledge and attitude changes did not capture these nuanced changes.

*Trial Registration* The parent feasibility trial was registered on January 01, 2018 with Clinical Trials Registry – India (CTRI), reg. no. CTRI/2018/01/011471, ref. no. REF/2017/11/015895. http://ctri.nic.in/Clinicaltrials/pdf_generate.php?trialid=21129&EncHid=&modid=&compid=%27,%2721129det%27..

**Supplementary Information:**

The online version contains supplementary material available at 10.1186/s13033-021-00453-3.

## Background

Addressing the care gap for children in need of mental health services is a crucial global health challenge, particularly in low and middle income countries (LMICs). In high income countries (HICs), 20% of children in need of mental health services receive them, already a substantial gap; in India, the LMIC study site, the gap is even wider, with fewer than 1% of children in need of mental health services receiving them [1–4].

The lack of trained mental health personnel is a significant barrier to increasing access to children’s mental health care in LMICs [5]. Alternative models of mental health care, such as task-shifting, can play an important role in bridging the care gap [5]. In a task-shifting model, professionals train and coach non-accredited individuals to deliver therapy [6]. Task-shifting mental health care to lay individuals in LMICs has been repeatedly shown to improve outcomes of adolescents and adults with mental illness [6–8]. Delivering task-shifted mental health care to children, however, has proven to be challenging [9]. Mental health care delivered to children must account for their consistently growing cognitive and emotion-regulation capabilities, requiring nuance, finesse, and experience [9].

Teachers in LMICs may be well-suited to be lay counselors who deliver indicated child mental health care [10–12]. They have relevant on-the-job experience in child development and hold the potential to impact child behavior through [1] their daily, consistent contact with children and [2] their ability to address the mental health needs of children in real time [10–15]. Further, schools are increasingly commonplace in LMICs, and school attendance rates have significantly improved since 2000, increasing the potential reach of teacher-delivered mental health care [9, 16]. Moreover, early evidence in HICs points to the ability of teachers to serve as narrow-spectrum lay counselors, demonstrating that trained teachers are able to use a small subset of mental health techniques to address specific symptoms related to Conduct Disorder [17, 18].

To date, however, teachers’ roles in child mental health care globally have been largely limited to mental health promotion or prevention centered on delivering classroom lesson plans to whole classes [18]. Teachers’ roles in delivering care may currently be narrow as generally they: (1) feel under-trained to teach and work with students with mental health concerns; (2) report being overburdened with their education duties and may lack the bandwidth to take on counseling tasks; and (3) may not view students’ mental health as their responsibility [10, 19].

Notably, teachers who receive appropriate training and supervision and acquire mental health experience in mental health promotion or prevention interventions in HICs have made gains in mental health knowledge, have had more positive attitudes towards mental health, and have improved self-efficacy in teaching students who need mental health support [17, 18, 20]. However, these studies largely involve teachers delivering prescribed, whole class lessons and their perspectives may differ from teachers who deliver indicated care to their students individually, a notable difference in duties [8, 18]. Moreover, HIC findings may not be generalizable to LMIC settings given differing levels of resources to support such programs [8, 18].

No published studies exist exploring teachers’ perceptions of serving as lay counselors and their knowledge of and attitudes towards mental health after training, supervision, and being a lay counselor for their school-aged students [8]. The paucity of studies is in part due to few published programs existing in which teachers act as lay counselors globally [8]. The one group that has studied teacher-delivery of youth mental health care for common mental disorders in an LMIC did not explore teacher knowledge of and attitudes towards mental health or perceptions of acting as lay counselor after playing this role [21]. These aspects of teachers’ experiences with student mental health may crucially underlie their ability and willingness to serve as lay counselor as part of filling the child mental health care gap. If these aspects remain unexplored, teachers may remain untapped as human resources for filling the care gap despite their unique capabilities to do so.

In this mixed methods study, we assess teacher knowledge of mental health after serving as lay counselors to their students, teacher attitudes towards mental health, and teacher perceptions of serving as lay mental health counselor. We examine crucial individual level factors that may underlie whether teachers can or will deliver indicated child mental health care in LMICs. We hypothesized that teachers would experience improvements in knowledge with training, supervision, and experience delivering care. Secondly, we hypothesized that teacher attitudes towards mental health would become more inclusive as evidenced by teachers demonstrating an understanding of the behaviors of children with mental illness based on psychological principles. Finally, we sought to explore teacher experiences and perceptions of serving as lay counselor as part of a novel children’s mental health care model.

## Methods

### Study design

This analysis sought to complete a secondary aim within a 2018 feasibility study of a teacher-led task-shifting system of children’s mental health care in low cost private (LCP) primary schools of the Darjeeling Himalayas, West Bengal, India. Using a mixed methods pilot pragmatic design, we assessed for the feasibility of teachers delivering mental health care to their students, the subject of another manuscript submitted for review. Throughout this study, we collected data, as described later, to address the secondary aim of understanding teacher knowledge of and attitudes towards mental health after being a lay counselor as well as their perceptions of being a lay counselor, the basis of this analysis.

### Setting

Darjeeling’s population of approximately 800,000 people is scattered in rural villages nestled in the Himalayan mountains [22]. The majority of residents are ethnically Nepali, a minority group within the State of West Bengal [22]. Daily wages are 120 Indian rupees (INR) (approximately $1.68 United States dollars (USD)) for tea plantation laborers, who make up the majority of workers (77%) [23, 24]. While a Darjeeling-specific prevalence of child mental illness has not been published, prevalence rates in a nearby rural area, also in West Bengal, estimated child psychiatric morbidity at 33% [2]. Unpublished observations from the authors’ needs assessment in 2017 revealed that there are 3 counselors and 1 general psychiatrist to address the mental health needs of approximately 100,000 youth. The demographics of teachers in Darjeeling, whether LCP or government, have not been published. One study aggregates LCP and government teacher demographics across all provinces in India. Compared to government school teachers, LCP school teachers tended to be younger (29.61 years versus 40.28 years), more likely to be college graduates (49% versus 39%), and less likely to hold teaching certificates (28% versus 80%) [25].

### Participants

To target schools enrolling children with poor access to care, LCP schools were considered eligible if they were located in rural Darjeeling, did not receive aid from the government, enrolled students from families with average daily incomes of $10 USD or less, and had total annual fees of $180 USD or less [24]. Of the 17 eligible schools contacted, 11 agreed to participate and 5 were chosen based on further inclusion criteria of having a population of 50 or more students to more accurately approximate prevalence rates of the general population. From these 5 rural LCP schools, 327 participants were enrolled. Eligible teachers were employed at enrolled schools, had 1 or more years of teaching experience (to avoid their perceptions and capabilities being tied to learning how to perform their primary duties as a teacher), were 18 years or older, and were not being investigated for or convicted of child maltreatment.

The full study sample consisted of 19 teachers, 36 children selected for mental health support and their 36 parents or guardians, and the remaining 236 children within teachers’ classrooms not selected for mental health support. Twenty-three teachers consented to participate in the study and completed the training, and 19 completed the full activities of the intervention. Completed mental health attitudes surveys from 15 teachers, completed mental health knowledge summative assessments from 14 teachers, and completed semi-structured interviews with 17 teachers were analyzed.

### Procedures

Teacher training consisted of 10 days of learning to deliver evidence-based children’s mental health care, including identifying children in need of mental health services, observing children’s behaviors through a basic functional behavior assessment, creating iterative behavior plans, and delivering Cognitive Behavior Play Therapy (CBPT) to students as part of the behavior plan [26]. After training and under the guidance of study staff, each teacher selected 2 students to work with whom they felt were in most need of mental health services. This pragmatic limitation was based on teacher feedback during intervention piloting where they expressed that 2 students each was a feasible caseload given their other responsibilities [27]. Teachers spent 2–3 months observing students and completing a basic functional behavior assessment on them. Afterwards, they developed a behavior plan to guide their interactions with students, including changing classroom environments and practices, setting limits, and completing CBPT work. They worked therapeutically with the students for the remainder of the school year (~ 6–8 months). A psychiatric social worker with child mental health expertise delivered the training, collected data on teacher mental health knowledge and attitudes and perceptions of their role as lay counselor, and provided twice monthly supervision to teachers.

## Measures

### Quantitative assessments

#### Teacher knowledge summative assessment

Teacher knowledge was assessed in summative assessments consisting of two written case vignettes highlighting the mental health struggles of children as covered in the training (Additional file 1). Each vignette focused on a theoretical child with a description of the child’s behaviors and interactions with others in class. Descriptions of the basic functional behavior assessment and behavior plan completed by the theoretical child’s teacher were then provided. Developed to align with training and intervention content, the summative assessment was designed to evaluate teacher knowledge of behavior theory, basic functional behavior assessment, and behavior plans as applied to the cases in the vignettes.

Participants were asked to answer 5 multiple choice questions, fill in 2 blank charts, and complete a matching column associated with the first vignette and 4 multiple choice questions related to the second vignette. Example multiple choice questions, one from each vignette, are as follows: (Vignette 1) What trigger immediately precipitates Sagar’s pushing of Rupak? (circle one); (Vignette 2) How did the breaks change Nishant’s environment and help Nishant decrease his outburst frequency? (circle all that apply). For the purposes of scoring the measure, only the 9 multiple choice answers were scored. The matching column and the filling in of two blank charts were included in the assessment to serve as a baseline comparison for programmatic qualitative monitoring and evaluation and were not included in scoring. The summative assessment scores for the measure could range from 0–9, with partial points given for a partially correct answer. Scores and feedback were not provided to teachers. Providing feedback was thought to possibly influence later test scores. Instead, study staff worked with individual teachers in supervision throughout the year to address deficiencies seen in summative testing. The assessment was available in Nepali and took 30 min to complete. It was administered at 3 time points: pre-training (PRE), post-training (POST), and intervention year-end (INT) (coinciding with academic year-end, approximately 6–8 months after POST).

Vignettes have been commonly used to test teacher knowledge and thought processes, specifically as they relate to student mental health; they mimic real world scenarios and thus are able to assess for the practical application of teachers’ knowledge of student mental health [28]. The vignettes in this study were unstandardized but modeled after those used to measure teacher mental health knowledge in other studies [28–30]. They were crafted to align with the training curriculum and content as well as the local cultural context. The vignettes were created by a panel of child mental health experts who agreed on the correct responses to each vignette’s questions. Local study staff with child mental health expertise then reviewed the vignettes to ensure relevance to the local context and also agreed on correct responses. The vignettes were then field tested for validity.

#### Teacher survey on mental health attitudes, self-perceptions of mental health knowledge, and perceptions of serving as lay counselor

Teacher’s mental health attitudes, self-perceptions of their knowledge, and perceptions on serving as lay counselor were assessed using a study specific survey (Additional file 2). The un-standardized survey was created based on a review of the literature detailing related surveys [10, 31, 32]. It was designed to capture 3 categories of interest: teachers’ self-perception of mental health knowledge, their mental health attitudes, and their perceptions of serving as lay counselors. Subcategories (Knowledge, Intent, Behavior, Job, Intervention, and Barriers) were used to further classify questions based on different aspects of the larger categories. Questions from “Knowledge” and “Barriers” mapped onto self-perceptions of knowledge; “Job” and “Behavior” onto attitudes; and “Job”, “Intent” and “Intervention” onto perceptions of serving as lay counselor.

Example questions from each category are as follows. (1) Knowledge: I am effective in assessing students with the following behavioral health concerns—disruptive behavior. (2) Intent: I intend to interact one on one with students with behavioral health concerns. (3) Behavior: I believe that the context or environment a person is in can influence the person’s behavior. (4) Job: Interacting one on one with students with behavioral health concerns to help manage their behavior is an important part of my job. (5) Intervention: Assessing students with behavioral health concerns is up to me. (6) Barriers: My lack of knowledge about behavioral health issues keeps me from assessing students with behavioral health concerns.

The survey consisted of 49 items with a five-point Likert scale, with answer options of: strongly agree, moderately agree, neither agree nor disagree, moderately disagree, and strongly disagree. We calculated separate scores for each of the 6 categories, as well as an overall score. A lower score indicated teachers were positively influenced by serving as a lay counselor within the system of care. The survey was available in Nepali and required 10 min to complete. It was administered PRE, POST, and INT. The assessment and survey were translated from English to Nepali, back-translated, and adjusted for accuracy after being compared with the original versions.

The survey items were based on a review of the literature detailing related surveys and designed to align with the social context [10, 31–34]. They were reviewed by a panel of experts in pediatric school health interventions for feedback on question coverage of categories and subcategories, relevance of questions, and any missing aspects relevant for understanding teacher knowledge, attitudes, and perceptions. Local study staff with child mental health expertise then reviewed the questions to ensure relevance to the local context. The survey was then field tested for validity.

### Qualitative assessment

At the INT time point, we conducted semi-structured interviews with 17 of 19 teachers to assess: intervention feasibility, acceptability, and impact; knowledge of and attitude towards mental health after being a lay counselor; teachers’ perceptions of serving as lay counselor; and ideas for improving the intervention. Interview guides were iteratively developed around themes of feasibility, acceptability, and impact and then finalized with the research team and program staff. Questions were open-ended to allow teachers to direct the flow of the interview (Additional file 3). Trained research assistants based in Darjeeling facilitated the interviews in Nepali. They recorded field notes to complement transcripts. Interviews were audio-recorded and then transcribed and translated into English by an independent translator. A study staff member reviewed transcriptions and translations for accuracy.

### Data analysis

#### Quantitative

Demographics of the teachers who completed surveys and assessments PRE (which were all teachers who initially enrolled in the study), those who completed the quantitative follow-up INT, and those who completed the interview INT were compared. The independent sample *t* test was used for continuous variables, χ^2^ for categorical variables, and Spearman’s Rank Correlation for ordinal variables.

Teacher knowledge summative assessment means were compared PRE to POST, POST to INT, and PRE to INT using the paired *t* test via ANOVA, with p < 0.05 considered significant. Cohen’s d was calculated to quantify potential effect sizes. Means of teacher mental health attitudes survey scores were evaluated using the paired *t* test, comparing PRE to POST score, POST to INT scores, and PRE to INT scores. We adjusted the significance level to be p < 0.0024, starting with a base p-value of p < 0.05 and adjusting it for 21 comparisons. SAS version 9.4 (Cary, NC) was used for all data analysis [35].

### Qualitative

The semi-structured interviews from 17 teachers were analyzed with the goal of qualitative description. An inductive content analysis approach was pursued using the software ATLAS.ti version 8.4.15, 2019 for analysis [36, 37]. Two independent analysts iteratively coded the data using the template coding style as per Crabtree and Miller [37, 38]. Each analyst coded all interviews. Group consensus was used to resolve coding discrepancies. Codes were coalesced to identify emergent themes alongside key supporting quotations. Results of the analysis were linked to the aims of understanding teacher knowledge of and attitudes towards mental health after acting as lay counselors as well as teachers’ perceptions of serving as lay counselor.

## Results

### Demographics

Teachers who completed quantitative measures PRE were 74% female. Those who additionally completed INT knowledge summative assessments were 71% female, while teachers who completed INT mental health attitudes surveys were 73% female. Teachers who completed interviews were 82% female. Teachers from scheduled caste or tribe, officially recognized groups of historically disadvantaged peoples by the Government of India, ranged from 30%–36%. The group of teachers who first enrolled in the study were not statistically different from those who completed either quantitative assessment or from those who completed the interview (Table 1).

**Table 1:** Teacher demographics

	Completed training and pre-training attitudes survey and knowledge summative assessment (PRE) (N = 23)^c^	Completed knowledge summative assessment post-training (POST) and at end of intervention (INT) (N = 14)^c^	Completed attitudes survey post-training (POST) and at end of intervention (INT) (N = 15)^c^	Completed semi-structured interview at end of intervention (INT) (N = 17)^c^
Continuous variables	Mean (range)	Mean (range)	Mean (range)	Mean (range)
Age in years	27.6 (21.0–39.0)	28.9 (22.0–39.0)	28.6 (22.0–39.0)	27.9 (21.0–39.0)
Years Teaching at Current School	4.17 (1.0–17.0)	4.9 (1.0–17.0)	4.9 (1.0–17.0)	4.8 (1.0–17.0)
Years Teaching Total	4.65 (1.0–17.0)	5.7 (1.0–17.0)	5.6 (1.0–17.0)	5.4 (1.0–17.0)

Categorical variables	N (%)	N (%)	N (%)	N (%)
Gender = Female	17 (74%)	10 (71%)	11 (73%)	14 (82%)
Member of Scheduled Caste/Tribe^a^	7 (30%)	5 (36%)	5 (33%)	6 (35%)
Language^b^				
Nepali	22 (96%)	13 (93%)	14 (93%)	16 (94%)
Bengali	2 (9%)	0 (0%)	0 (0%)	0 (0%)
English	21 (91%)	13 (93%)	14 (93%)	17 (94%)
Hindi	16 (70%)	9 (64%)	9 (60%)	10 (58%)
Other	0 (0%)	0 (0%)	0 (0%)	0 (0%)
Level of Education				
Some Primary	1 (4%)	0 (0%)	0 (0%)	0 (0%)
Finished Primary	0 (0%)	0 (0%)	0 (0%)	0 (0%)
Some Secondary	2 (9%)	2 (14%)	2 (13%)	2 (12%)
Finished Secondary	2 (9%)	0 (0%)	0 (0%)	1 (6%)
Undergraduate or Higher	18 (78%)	12 (86%)	13 (87%)	14 (82%)
Has Formal Training in Education	4 (17%)	4 (29%)	4 (27%)	4 (24%)
Has a Teaching Certificate	3 (13%)	3 (21%)	3 (20%)	3 (18%)
Class Levels Taught^b^				
Class I (Kindergarten)	12 (52%)	7 (50%)	7 (47%)	8 (47%)
Class II (1st Grade)	17 (74%)	9 (64%)	10 (67%)	12 (71%)
Class III (2nd Grade)	17 (74%)	9 (64%)	10 (67%)	12 (71%)
Class IV (3rd Grade)	19 (83%)	11 (79%)	12 (80%)	14 (82%)
Class V (4th Grade)	19 (83%)	10 (71%)	11 (73%)	13 (76%)
Class VI (5th Grade)	14 (61%)	8 (57%)	8 (53%)	10 (59%)
Class VII (6th Grade)	6 (26%)	6 (43%)	6 (40%)	5 (29%)
Additional School Responsibilities				
Yes	6 (26%)	5 (36%)	5 (33%)	5 (29%)
Sports	3 (50%)	3 (60%)	3 (60%)	3 (60%)
Cultural	1 (17%)	1 (20%)	1 (20%)	1 (20%)
Accounting	1 (17%)	1 (20%)	1 (20%)	1 (20%)
Typing	1 (17%)	0 (0%)	0 (0%)	0 (0%)
Other Employment				
Yes	15 (65%)	10 (71%)	10 (67%)	10 (59%)
Housework	11 (48%)	8 (57%)	8 (53%)	9 (53%)
Selling things/running a shop	1 (4%)	1 (7%)	1 (7%)	1 (6%)
Farming/agricultural	4 (17%)	3 (21%)	3 (20%)	2 (12%)
Work on tea garden	0 (0%)	0 (0%)	0 (0%)	0 (0%)
Transport (driver, etc.)	0 (0%)	0 (0%)	0 (0%)	0 (0%)
Health worker	0 (0%)	0 (0%)	0 (0%)	0 (0%)
Manual labor	0 (0%)	0 (0%)	0 (0%)	0 (0%)
NGO	1 (7%)	0 (0%)	0 (0%)	0 (0%)
Tour Guide	1 (7%)	1 (10%)	1 (10%)	1 (6%)
Tutoring	5 (33%)	4 (40%)	4 (40%)	5 (29%)

^a^Scheduled Caste and Scheduled Tribe are standard terms used in Indian demographic surveys referring to officially recognized groups of historically disadvantaged peoples by the Government of India and State of West Bengal

^b^Sum is greater than 100% due to individuals speaking multiple languages, teaching multiple grade levels, or having multiple side jobs

^c^p-value < 0.05 was used for comparison between those who did and did not complete the final study activities, using Fisher’s exact test for categorical variables, and Student’s t-test for continuous variables. No demographic variables were significantly different across time periods

### Quantitative results

Teacher knowledge as measured by the study-specific summative assessment across all teachers did not show any changes PRE versus POST, POST versus INT, and PRE versus INT (Table 2). Examined descriptively, 7 of the 14 teachers had sustained higher INT summative assessment scores compared to their pre-training scores, demonstrating sustained improvement over time. Six teachers had lower INT summative assessments scores compared to PRE and one teacher had the same PRE and INT score.

**Table 2:** Mean scores for pre, post, and post-intervention for knowledge summative assessments

	PRE N = 23	POST N = 23	INT N = 14	PRE-POST comparison p-value^a^, Cohen’s d	POST-INT comparison p-value^a^, Cohen’s d	PRE-INT comparison p-value^a^, Cohen’s d	ANOVA
	Mean (SD)	Mean (SD)	Mean (SD)				
Total score	5.61 (1.03)	5.66 (1.38)	5.30 (1.54)	0.85, 0.04	0.78, 0.25	0.77, 0.24	0.70

^a^t-test

^significant if p < 0.05; no comparisons were significant

**Fig. 1:**
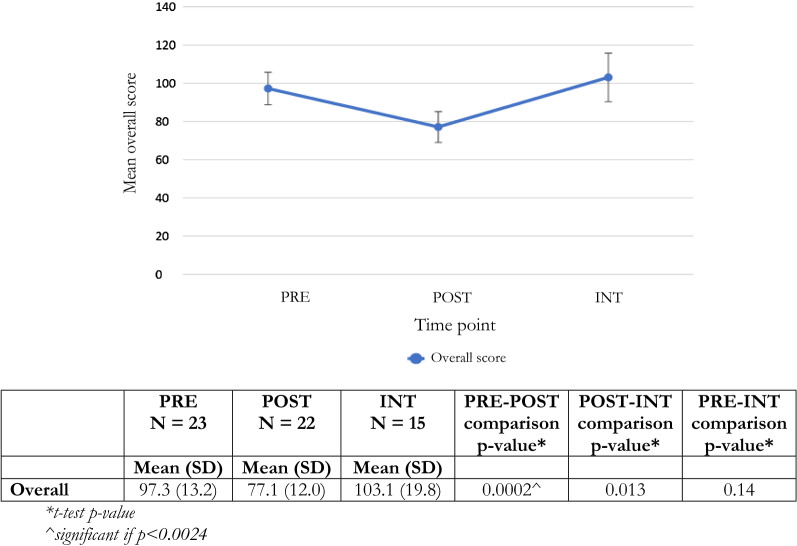
Mean overall scores, 95% confidence intervals and t-test comparisons for pre, post, and post-intervention for attitudes, knowledge, and perceptions surveys

**Fig. 2:**
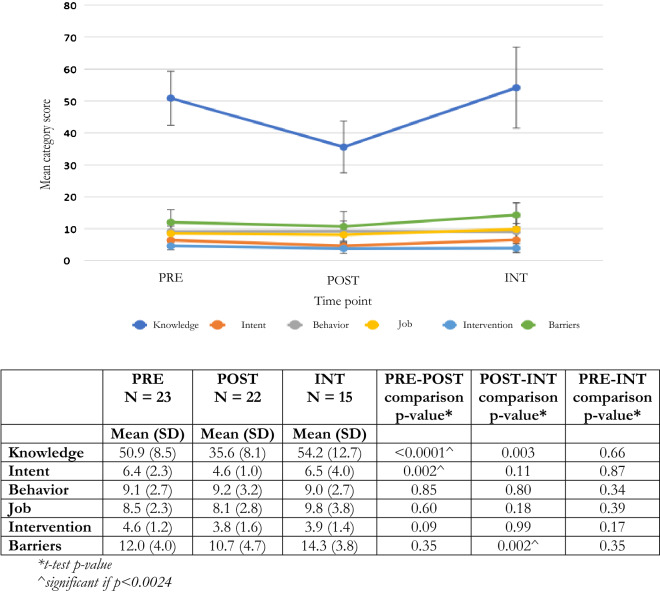
Mean subcategory scores, 95% confidence intervals, and t-test comparisons for pre, post, and post-intervention for attitudes, knowledge, and perceptions surveys

On the survey assessing mental health attitudes, knowledge, and perceptions of serving as lay counselor, teachers showed improvements in their overall scores POST compared to PRE, with lower POST training scores on the study-specific survey (Fig. 1). At INT, teachers’ scores returned to the same level as their pre-training scores, indicating that teachers’ mental health knowledge and attitudes and perceptions of serving as lay counselor reverted to pre-training levels. Individual subcategory scores either followed a similar pattern as the overall score or remained the same on average PRE, POST, and INT (Fig. 2).

### Qualitative results

Predominant themes and representative quotes from semi-structured interviews conducted at INT are displayed in Table 3. Themes and quotes reported were those related to teachers’ mental health knowledge and attitudes and perceptions of serving as lay counselor.

**Table 3:** Themes and representative quotes from teacher semi-structured interviews

Question Category	Theme	Quote
Knowledge	Ability to identify children in need	*“I thought of one child and when the time came to identify one who needed help, I ended up choosing the child. In the training, we had learned to identify the qualities of a withdrawn child. This child possessed these qualities”*
	Ability to use program tools to support children	*“There was this child in the primary section that was never active in class. The child wasn’t eager to join or participate in any of the activities and we assumed that was her nature. We had never before coaxed her to join in. We thought this is how she worked. But after the training we started to coax her and started to give her positive words of encouragement, and we told her that she is such a capable and good girl and that she can do it. This approached worked because the child started to participate in the activities at school and even wanted to be part of the small drama that we wanted to have and the child came in front of all these people and spoke very well. The child did not feel shy or scared”* *“I used tools to calm them down and also referred to the physical coping strategies such as taking long deep breaths to help them calm down and cope with the situation”* *“We started to give the students stars according to their homework or class work performance and when the child got stars it motivated and encouraged them to work harder and made them realise that they can also come up to the level of the other students and there was progress in their performance too”* *“The child would not mix about and talk with the other children, but when I made the child sit with the brainy students the child started to work better in class and started to feel that he/she could also do better like the brainy students”* *“I told the class that each one of them would be called for an interview one by one. I sat them down and made them draw the smiley faces. I asked them to make three faces, the happy, angry faces and started to call them privately one by one. I did not call on her at first. She was probably the 3*^*rd*^* or the 4*^*th*^* student to come for the pretend interview in which we were able to talk and I got to know about her emotions too. In this way I got my work done and all the kids were happy too”*
	Change in self-efficacy	*“Earlier we would very easily punish the child without trying to find out the reason for his/her actions. But now we know that there are issues that lead a child to do that mischief or there is a reason why the child is behaving in that manner. We are much more aware and know better than to punish, scold, or beat up the child”* *“I started to understand that sometimes you need to talk and have a conversation with the student and explain why that action or behavior is unacceptable and how he/she should change and correct the wrongs in his speech and action. Many a times such talks actually work better with them than punishment”*
	Challenges	*“I was a bit unclear and unsure on how to use the emotional thermometer, so I didn’t use it”* *“The parents of the children who live nearby do make time to come but the ones who are far away don’t have the time to come and neither do we have the time to go all the way to their houses to conduct the one on one. Besides this, we are also very uncomfortable to hold one on one with the parents”* *“Sometimes it felt that nothing good can happen or be done to the child and the child wouldn’t improve. But we had monthly visits by (study staff) and she would look at our work and tell us if this approach didn’t work, the next month we should try the other approach”*
Attitudes	Changes in beliefs about mental health	*“Had it not been for the training I would probably have called him (the student) a thief… but the training taught me better”* *“Before the training we would assume that the child was not interested and we wouldn’t bother going over to them and finding out why they aren’t showing any interest like the rest. We would tell them if they do the work it’s for their own good and if they do not, then it’s their loss. But now we have taken the training, we have come to understand that there is a reason for the withdrawal. Instead of treating them like outcastes, we now go to them and engage them in conversation and try to find out what is bothering them”*
Perceptions of serving as a lay counselor	Serving as lay counselor was acceptable	*“We would like to thank you for giving us the skills and trainings through which our children have been able to improve, and we would like to get many more such trainings”* *“The program should not stop; it needs to continue and not just in our area… there must be a lot of other such schools in other rural areas, and they would most certainly need this kind of program.”*
	Need for more support	*“I felt we would really need additional training to teach us how to do all these things because I feel the training was done at such a short span of time that we didn’t get enough time”*

#### Mental health knowledge

A majority of teachers expressed retaining the knowledge they learned from the training and supervision (Table 3). Many discussed grasping mental health concepts, behavior analysis and behavior plan elements taught in the training. Regarding specific CBPT techniques, teachers rarely discussed them in name. A few teachers showed an understanding of CBPT techniques as indicated by discussing single tools they regularly used. More often, teachers reported incorporating CBPT concepts into the knowledge transfer process, their primary duty (see illustrative quotes in “Ability to use program tools to support children” in Table 3) [39]. As a result, they used the process of knowledge transfer not just to transfer knowledge, but also with the aim of improving the mental health status of their students. They also discussed tailoring their classroom interactions with and classwork for the targeted students to better meet the students’ needs, such as providing in-class accommodations for their schoolwork. Teachers additionally discussed the novelty and usefulness of the basic therapeutic principles of relationship-building and asking open-ended questions. Their described use of these principles was most often during instructional time, breaks in their schools’ schedules, or before or after school.

A few teachers felt they needed more time to learn the training material to better grasp and apply mental health knowledge (Table 3). Some teachers expressed uncertainty as to how to use specific skills taught during the training. However, several teachers described supervision as a mechanism to later grasp and retain knowledge from the training. A few teachers expressed wanting more supervision sessions to guide their care.

Teachers almost unanimously expressed a greater self-efficacy in managing the mental health of students in their classrooms, an indication of applied knowledge retention (Table 3). Teachers stated decreasing use of the following practices while working with students with mental health struggles: (1) using only an authoritative voice in the classroom, (2) using physical punishment to motivate the children, and (3) speaking with children “roughly” or “rudely”.

#### Mental health attitudes

Teachers overall expressed progression towards inclusive mental health attitudes, beliefs, and practices (Table 3). Most expressed newly understanding students’ behavior through a psychological lens after serving as lay counselor. Teachers also reported understanding the importance of mental health within their classrooms for student learning. Some discussed adjusting their teaching styles to better accommodate their students with mental health struggles.

#### Perceptions of serving as lay counselor

A majority of teachers indicated a willingness to serve as lay counselor (Table 3). As a whole, teachers perceived their duties as lay counselors to be the following: student selection for mental health services, conducting basic functional behavior assessments of the chosen students, creation of a behavior plan that included delivery of CBPT, implementing the behavior plan, modifying the behavior plan based on the student’s evolution of symptoms and/or efficacy of the initial plan, and involving parents in the task-shifted care of the students. These perceptions were in line with those we outlined in their training. Several teachers showed enthusiasm at the opportunity to continue to be a lay counselor within the program. Many discussed their regular use of mental health skills learned and changes to their own discipline practices as an indicator of willingness to serve as a lay counselor. Some expressed a desire to learn more skills to improve their practice. Some further requested for the program to expand to other schools given the need they felt the program filled in their school.

Some teachers had concerns with certain aspects of the program (e.g., *“The thing that didn’t quite work is the one where we give stars to the students for their work,”*) or the pace of progress in children (e.g., *“I cannot say it’s extremely effective and there had been a lot of changes… However, I have seen and observed that are some changes in them [students] and I even get to hear from the parents that there has been changes in the child.”*). Overall, no teachers broadly criticized the program or their role as lay counselor.

## Discussion

This study contributes to the emerging literature on task-shifting children’s mental health care to schools as part of ongoing efforts to bridge the wide care gap, particularly in LMICs. Most studies show teacher involvement in school mental health confined to promotion or prevention curriculum delivery, limiting publications of assessments of teacher knowledge and attitudes to those with a different, perhaps less intensive focus [8, 40]. Only one group has explored whether teachers can be leveraged to deliver indicated care; the youth who received the study’s teacher-delivered care did not demonstrate changes to their mental health status [8, 21]. As previously discussed, the role of individual-level factors and whether they were modifiable were not explored as part of that study’s negative results [21, 41, 42]. By contrast, this study assesses how training, supervision, and experience can influence individual-level teacher factors that may underlie teachers’ ability to deliver effective indicated child mental health care. It uniquely contributes to an exploration of whether teachers may be willing or able to deliver indicated care to fill the care gap.

The results present a complex picture of the influence that mental health training, regular supervision, and working as a lay counselor have on teacher mental health knowledge and attitudes and their perceptions of serving as lay counselor. Findings from the qualitative data are discordant with the results from study-specific quantitative forms. Teacher knowledge as measured by a study-specific assessment largely did not appear to change when comparing PRE, POST, and INT. Semi-structured interviews at INT, though, revealed that teachers grasped knowledge from the training and supervision and further changed their own behavior and teaching practices, a demonstration of knowledge retention. Similarly, teachers’ mental health attitudes on a study-specific survey improved POST compared to PRE, but at INT appeared to revert to PRE levels. However, in semi-structured interviews at INT, a majority of teachers expressed that they underwent significant changes in their attitudes towards mental health and provided examples of being more inclusive of students with mental health struggles. Moreover, a majority of teachers expressed positive perceptions of being a lay counselor, expressed interest in continuing to serve as lay counselors, and encouraged study staff to expand the program to other schools.

Use of semi-structured interviews allowed teachers to share their experiences with little prompting, leading to insights that may not have been clear to ask about with closed-ended questions. By contrast, the study-specific summative assessment and survey quantitatively evaluated the mental health knowledge and attitudes of teachers with questions determined a priori. Scant literature published exploring the mental health knowledge and attitudes of teachers who have served as lay counselors was available to guide question formation. Instead, these questions were based on the working hypothesis for the intervention’s mechanism of change that teachers grasped and retained knowledge, shifted attitudes, and changed their daily practice the way mental health trainees in a professional mental health training program would, with mastery of every detail and nuance of behavior theory and mental health care delivery [8, 21]. By contrast, the semi-structured interviews revealed that teachers grasped, retained, and used mental health knowledge and changed attitudes towards mental health that were relevant to the way in which mental health techniques could be incorporated into teachers’ primary duty of educating children, such as increasing empathy, building individual relationships and trust, and utilizing classwork as a means to improving their students’ mental health status.

To illustrate this contrast, we first examine the summative assessment (that measures teacher knowledge), specifically Questions 1 and 2 (Additional file 1): (1) “what trigger immediately precipitates Sagar’s pushing of Rupak?” and (2) “what is the root cause of Sagar’s inappropriate behavior the first morning?”. Testing key aspects of behavior theory, these two questions ask teachers to distinguish between the immediate trigger of a behavior (antecedent) and the root cause that may underlie the behavior though may not be the immediate cause of it [26]. A representative quote provides insight into how teachers may have come to understand antecedents and root causes (from Table 3).

“There was this child in the primary section that was never active in class. The child wasn’t eager to join or participate in any of the activities and we assumed that was her nature. We had never before coaxed her to join in. We thought this is how she worked. But after the training we started to coax her and started to give her positive words of encouragement, and we told her that she is such a capable and good girl and that she can do it. This approach worked because the child started to participate in the activities at school and even wanted to be part of the small drama that we wanted to have and the child came in front of all these people and spoke very well. The child did not feel shy or scared.”

Without distinguishing between trigger and root cause, this teacher tailored the delivery of mental health support to simultaneously address both the implied trigger (overwhelming school activities) and implied root cause (a belief that she (student) was incapable of completing the activities, perhaps due to social reasons). This teacher’s report of how intervention tools were used was similar to others’ reports. Distinguishing between the two types of preceding events was seemingly less relevant and instead implementing a school-based solution to improve the child’s mental health was prioritized. The mastery of specific details of therapy delivery, all of which would be relevant to a mental health practitioner, was evidently less pertinent to teachers delivering task-shifted mental health care. Thus, their ability to answer study-specific summative assessment questions that assessed their knowledge on these details may have been accordingly less accurate.

Similarly, survey questions assessing teacher perceptions of mental health knowledge and attitudes presumed that teachers would deliver care with mastery of and use of all tasks similar to a mental health professional. Here, we further explore a specific example, looking at how the survey assessed teachers regarding the task of having regular one on one sessions with the student (Additional file 2) and comparing it with teacher sentiments on this task as captured in qualitative interviews (Table 3).

To gauge teacher sentiments on one on one interactions with students, teachers were asked their level of agreement or disagreement with several statements, one of which was: “I interact one on one with students with behavioral health concerns to help manage their behavior often”. In interviews, some teachers discussed adapting intervention tasks into the knowledge transfer process in a way that could have led them to disagree with this statement. For example, a few teachers reported reconfiguring their one-on-one time to instead be class-wide activities. The teacher who relayed the following (as in Table 3) may not have classified such time with the child as “one on one”.

“I told the class that each one of them would be called for an interview one by one. I sat them down and made them draw the smiley faces (on an emotion thermometer). I asked them to make three faces, the happy, angry faces and started to call them privately one by one. I did not call on her at first. She was probably the 3rd or the 4th student to come for the pretend interview in which we were able to talk and I got to know about her emotions too. In this way I got my work done and all the kids were happy too.”

The two examples illustrate how teachers’ completion of intervention tasks differed enough from our expectations to potentially underlie discordant quantitative and qualitative results. As these expectations were the foundation from which we created the summative assessment and survey, the results of these quantitative measures, that teacher knowledge and attitudes seemingly reverted to PRE levels at INT, are accordingly at odds with the qualitative results.

At the center of the seeming discordance between qualitative and quantitative results is the freedom afforded to the teachers to adapt the delivery of the intervention to their needs. While teachers varied in their use of tools outside of the classroom and the timing of the use, most teachers commonly reported the incorporation of basic therapeutic interactions into classroom instruction time by using mental health techniques as part of their knowledge transfer process. In other words, teachers reported modifying instructional methods as the “therapy” to improve the mental health of their students. Teachers in the study may have organically created a new form of therapy, and perhaps even an emerging model of care, through an unexpectedly consistent application across teachers of therapy tools to the knowledge transfer process. Here, we coin the term “education as mental health therapy” to describe this phenomenon.

Unpublished observations of teacher fidelity to the intervention, the subject of another manuscript submitted for peer review, corroborate that teachers were modifying their instructional methods to incorporate mental health therapy techniques. Study staff observed, for example, teachers praising students for completing in-class assignments to provide evidence against automatic thoughts students had held that they were incapable of completing them. Through this incorporation, the process of knowledge transfer became therapeutic to the child, specifically targeting improvement of the child’s mental health and going further than the typical goal of instruction (knowledge transfer).

Notably, we believe our unpublished observations support that teachers were able to deliver the intervention with fidelity (manuscript submitted for peer review). The intervention is not a traditional, manualized therapy; instead, it provided teachers with a menu of therapeutic techniques to choose from for their work with the selected child and for which they were given freedom of choosing when to use them. Teachers were observed to deliver the intervention with fidelity since their decision to incorporate these techniques into instructional time was within what was expected. The novelty we observed, however, was that many of the teachers chose to deliver the intervention *primarily* through incorporating therapeutic techniques into the knowledge transfer process, in line with the qualitative results reported here. Still, this finding was not particularly notable in our fidelity findings as they remained in line with general expectations.

Central to an “education as mental health therapy” potential model of care is the teacher’s adapting of pedagogy to the needs of different learners, considered to be standard throughout education [43, 44]. In general education, adjusting teaching techniques to individual students is considered ideal practice [43, 44]. In special education in HICs, adjusting teaching techniques to the special needs of students identified as exceptional is an essential teaching practice [43, 44]. For example, a similar term, “education as therapy”, exists in special education and refers to a way for teachers to think about how the education they deliver can build the deficit academic skills of a child with disabilities using the knowledge transfer process [45]. Moreover, “educational therapy” is a process in which teachers deliver personalized, remedial instruction to children with learning difficulties that also aims to build the children’s confidence and self-esteem, seen as a form of therapy the way practitioners of occupational therapy or speech therapy remediate deficit skills in those respective domains [45]. The primary end goal of educational therapy and education individualization is knowledge transfer, with teachers aiming to address students’ non-academic needs just sufficiently to allow for knowledge transfer; improving students’ mental health status is not a primary goal, if a goal at all [44]. In this study, by comparison, teachers use the knowledge transfer process with an end goal of improving students’ mental health outcomes.

Evidence in the literature points to a potentially stark difference between “educational therapy” and “education as mental health therapy”. Studies aiming to improve teachers’ ability to implement developmentally appropriate teaching practices and individualization through specific training have shown that academic outcomes improve, but mental health ones do not [12, 46]. By contrast, unpublished observations, the subject of a separate manuscript submitted for review, show that the children receiving care from their teachers in this study may improve in their mental health status. Teachers assessed student mental health status via the Achenbach System of Empirically Based Assessment (ASEBA) Teacher Report Form (TRF) [47]. Considered a gold standard, the measure is filled out by teachers and maps teacher impressions of a student’s mental health concerns onto validated categories of mental health challenges in children. On average and significant at the p < 0.05 level, children’s mental health scores at endline (n = 37) were reduced to a borderline level of mental health struggle (mean 60.09, standard error [SE] 4.98; borderline score range 60–63) compared to baseline clinical level scores (mean 77.51, SE 3.47; scores > 63 were considered clinical level), suggesting improvement in mental health status.

### Practice implications

An “education as mental health therapy” model of care relies on teachers shifting their professional practice to include mental health techniques. Teachers in this study reported notable shifts in their practice, including decreased use of (1) physical punishment, (2) authoritarian voices, and (3) speaking “roughly” or “rudely” with their students. These changes to practice have been difficult to instill broadly amongst teachers in India, serving as a potential barrier to teachers delivering care in this setting [48, 49]. Traditional teacher practices may be reflective of the attitudes of teachers in India towards mental health [48, 49]. Teachers in India have expressed mixed to negative attitudes towards children with behavioral struggles and their inclusion in the classroom [33]. In this study, teachers reported that, as a result of the training and supervision they received and having to act as a lay counselor, their attitudes changed and their practice evolved accordingly. Thus, an “education as mental health therapy” model may be possible with the appropriate training and supports in place to encourage teacher attitude shifts and subsequently practice change.

Notably, many teachers in the study did not have formal training in education (Table 1). It is possible that these teachers were more willing to be trained and serve as lay counselors as they may have been eager to learn skills in behavior management that they had not been formally taught. However, we found that most teachers in this study, whether formally trained or not, expressed similar sentiments in willingness to be trained to be and serve as lay counselors. They expressed that they had not received training on working with students with mental health concerns before and found the skills crucial to working confidently and effectively with these students, as exemplified by quotes in Table 3 under “Change in self-efficacy”. As teachers with formal training expressed similar views to those without formal training around willingness to serve as lay counselor, a wide range of teachers may be willing to serve as lay counselor more broadly.

By delivering care to children during their actual moments of struggle as part of a naturally occurring process in a child’s every day, “education as mental health therapy” deviates from the traditional office-based therapeutic sessions in which activities are solely focused on examining thoughts, feelings, and coping skills in isolation. The findings from this study indicate that teachers accordingly may not grasp and apply knowledge in the same way or have similar attitude changes as lay counselors who are delivering care in office-like models. They in turn highlight a potential limitation of teacher-delivered care overall—whether teachers have the time and capacity to deliver traditional one-on-one care to children in need of mental health services in the same way in which non-teacher lay counselors have been shown to do [6, 21]. However, findings from this study indicate that an alternative form of care, “education as mental health therapy”, may emerge as a teacher-delivered system of care that is in line with typical teacher duties while concurrently addressing students’ mental health needs. Whether this study’s findings are a precursor to an alternative, effective system of care warrants further investigation.

## Limitations

This study has a number of limitations. With a small, pragmatic sample, the study results are exploratory and not conclusive; further studies with larger sample sizes may be able to more conclusively answer whether teacher knowledge and attitudes change under similar programming. Moreover, the teachers in this non-randomized pilot may have been highly motivated and thus may not be representative of the teacher population as a whole. Further, there may be a demand characteristic at play, where teachers may have subconsciously inflated the reported, qualitative degree to which the intervention has changed their cognitions and behaviors. It is possible that these findings may not occur across a broader population of teachers acting as lay counselors or across teachers delivering the task-shifted care outside of observed research.

The lack of change in the quantitative measures of teacher knowledge and attitudes may have been due to the intervention design and not a mismatch between survey questions and teacher experience. As a few teachers indicated the need for more time in training and the perceived utility of more frequent supervision sessions, teachers may actually be able to deliver office-like care with more professional support than provided during the study. It is also possible that the quantitative measures were assessing knowledge and attitude change expected at the level of a trainee in a mental health professional program; expected answers may have been more granular and advanced than what would be expected for a lay counselor with other significant job duties to achieve under the current study conditions.

## Conclusion

After completing training, receiving supervision, and serving as a lay counselor, teachers in this study were able to grasp and retain mental health knowledge in a way that appeared to support their ability to deliver a form of task-shifted child mental health care. They expressed a positive change in their attitudes towards mental health and demonstrated favorable perceptions of serving as lay counselors. The qualitative and quantitative discordance in findings highlights the need for additional research using a larger sample size and asking questions that more accurately assess teacher practices. Such research could further validate results from this study and explore how teacher-delivered care may take structure. Findings from this study allude to a system of care, “education as mental health therapy”, that is closer in form to an educational intervention that concurrently addresses student mental health rather than the traditional office-based model of mental health.

Once robustly documented and understood as a system, further studies are recommended to assess whether an educational intervention as a system of care results in improved mental health outcomes for children in need of mental health care in LMICs, where the care gap is widest [1, 3]. Should children receiving care in an “education as mental health therapy” system show improved child mental health outcomes, such an alternative system of child mental health care may be sustainable. Teachers are existing human resources that could be leveraged to deliver care that could seamlessly be incorporated into their daily work, all occurring within an existing societal structure. Thus, in addition to exploring how training, supervision, and acting as a lay counselor may affect teachers’ mental health knowledge and attitudes, this study may document the beginnings of a novel alternative system of child mental health care with the potential to improve access significantly, potentially being available everywhere teachers teach.

## Supplementary Information

**Additional file 1:** Training summative assessment. This is the summative assessment used to assess teacher mental health knowledge PRE, POST, and INT.

**Additional file 2:** Attitudes, knowledge, and perceptions survey. This is the study-specific survey used to assess teacher mental health attitudes, self-perceptions of knowledge, and perceptions of serving as lay counselor.

**Additional file 3:** Classroom teachers semi-structured interview guide questions. This is the semi-structured interview guide questions used to interview teachers INT for qualitative data collection.

## Data Availability

The datasets generated and/or analyzed during the current study are not publicly available due to the connectedness of the Darjeeling community, the relatively small sample size of teachers included where families may be able to connect which children they know received services, and with mental health continuing to be stigmatized in the Darjeeling area. Participants did not agree to share their data publicly and they are NOT available from the corresponding author on request.

## Abbreviations

CBPT: Cognitive Behavior Play Therapy
HICs: High income countries
INR: Indian rupees
LMIC: Low and middle-income countries
LCP: Low cost private primary schools
USD: United States dollars
PRE: Pre-training
POST: Post-training
INT: Intervention year-end (coinciding with academic year-end, approximately 6–8 months after POST)

## References

[1] World Health Organization (2010). mhGAP intervention guide for mental, neurological and substance use disorders in non-specialized health settings.

[2] Banerjee T (1997). Psychiatric morbidity among rural primary school children in West Bengal. Indian J Psychiatry.

[3] Hossain MM, Purohit N (2019). Improving child and adolescent mental health in India: Status, services, policies, and way forward. Indian J Psychiatry.

[4] Burns BJ, Costello EJ, Angold A, Tweed D, Stangl D, Farmer EM (1995). Children's mental health service use across service sectors. Health Aff.

[5] Kakuma R, Minas H, van Ginneken N, Dal Poz MR, Desiraju K, Morris JE (2011). Human resources for mental health care: current situation and strategies for action. Lancet.

[6] Patel V, Weiss HA, Chowdhary N, Naik S, Pednekar S, Chatterjee S (2010). Effectiveness of an intervention led by lay health counsellors for depressive and anxiety disorders in primary care in Goa, India (MANAS): a cluster randomised controlled trial. Lancet.

[7] Patel V, Weobong B, Weiss HA, Anand A, Bhat B, Katti B (2017). The Healthy Activity Program (HAP), a lay counsellor-delivered brief psychological treatment for severe depression, in primary care in India: a randomised controlled trial. Lancet.

[8] Van Ginneken N, Tharyan P, Lewin S, Rao GN, Meera S, Pian J (2013). Non-specialist health worker interventions for the care of mental, neurological and substance-abuse disorders in low-and middle-income countries. Cochrane Database Syst Rev..

[9] Fazel M, Patel V, Thomas S, Tol W (2014). Mental health interventions in schools in low-income and middle-income countries. Lancet Psychiatry.

[10] Reinke WM, Stormont M, Herman KC, Puri R, Goel N (2011). Supporting children's mental health in schools: teacher perceptions of needs, roles, and barriers. Sch Psychol Q.

[11] Heisner MJ, Lederberg AR (2011). The impact of Child Development Associate training on the beliefs and practices of preschool teachers. Early Childhood Res Quar.

[12] Özler B, Fernald LC, Kariger P, McConnell C, Neuman M, Fraga E (2016). Combining preschool teacher training with parenting education: a cluster-randomized controlled trial.

[13] Auger RW (2004). The accuracy of teacher reports in the identification of middle school students with depressive symptomatology. Psychol Sch.

[14] Bradshaw CP, Buckley JA, Ialongo NS (2008). School-based service utilization among urban children with early onset educational and mental health problems: the squeaky wheel phenomenon. Sch Psychol Q.

[15] Snider MH, Fu VR (1990). The effects of specialized education and job experience on early childhood teachers' knowledge of developmentally appropriate practice. Early Childhood Res Quart.

[16] UNESCO. Progress in getting all children to school stalls but some countries show the way forward. Education for All Global Monitoring Report Policy Paper. 2014;14.

[17] Fairbanks S, Simonsen B, Sugai G (2008). Classwide secondary and tertiary tier practices and systems. Teach Except Child.

[18] Franklin CG, Kim JS, Ryan TN, Kelly MS, Montgomery KL (2012). Teacher involvement in school mental health interventions: a systematic review. Child Youth Serv Rev.

[19] Patel V, Kieling C, Maulik PK, Divan G (2013). Improving access to care for children with mental disorders: a global perspective. Arch Dis Child.

[20] Han SS, Weiss B (2005). Sustainability of teacher implementation of school-based mental health programs. J Abnorm Child Psychol.

[21] Shinde S, Weiss HA, Varghese B, Khandeparkar P, Pereira B, Sharma A (2018). Promoting school climate and health outcomes with the SEHER multi-component secondary school intervention in Bihar, India: a cluster-randomised controlled trial. The Lancet..

[22] Gorkhaland Territorial Administration. GTA Profile 2018. http://www.gta-darjeeling.org/node/285.

[23] Rai RP. Small farmers: locating them in “Darjeeling Tea”. Bag N, Bag A, Palini LMS, editor. Tea Technological Initiatives. New Delhi, India: New Delhi Publishing Agency; 2017. p. 19–38.

[24] Matergia M, Ferrarone P, Khan Y, Matergia DW, Giri P, Thapa S (2019). Lay field-worker–led school health program for primary schools in low-and middle-income countries. Pediatrics.

[25] Muralidharan K, Kremer M (2006). Public and private schools in rural India.

[26] Knell SM (1993). Cognitive-behavioral play therapy.

[27] Cruz CM, Lamb MM, Hampanda K, Giri P, Campbell M, Chowdhury B, Giardina AA, Gaynes BN, Matergia M (2021). Teacher nomination of school-aged children for mental health services in a low and middle income country. Global Health Action.

[28] Whitley J, Gooderham S (2016). Exploring mental health literacy among pre-service teachers. Exceptionality Education Int..

[29] Loades ME, Mastroyannopoulou K (2010). Teachers’ recognition of children’s mental health problems. Child Adolesc Mental Health.

[30] Green JG, Guzmán J, Didaskalou E, Harbaugh AG, Segal N, LaBillois J (2018). Teacher identification of student emotional and behavioral problems and provision of early supports: a vignette-based study. J Emot Behav Disord.

[31] Chorpita BF, Becker KD, Daleiden EL (2007). Understanding the common elements of evidence-based practice: misconceptions and clinical examples. J Am Acad Child Adolesc Psychiatry.

[32] White JL, Kratochwill TR (2005). Practice guidelines in school psychology: Issues and directions for evidence-based interventions in practice and training. J Sch Psychol.

[33] Sharma U, Moore D, Sonawane S (2009). Attitudes and concerns of pre-service teachers regarding inclusion of students with disabilities into regular schools in Pune. India Asia Pac J Teacher Education.

[34] Parasuram K (2006). Variables that affect teachers’ attitudes towards disability and inclusive education in Mumbai. India Disability Society.

[35] SAS Institute Inc. SAS/ACCESS^®^ 9.4 Interface to ADABAS: reference. Cary, NC: SAS Institute Inc.; 2013.

[36] ATLAS.ti Scientific Software Development GmbH. ATLAS.ti version 8.4.15. Berlin: ATLAS.ti Scientific Software Development GmbH; 2019.

[37] Thomas DR. A general inductive approach for qualitative data analysis. 2003.

[38] Crabtree BF, Miller WL (1999). Doing qualitative research.

[39] McKeough A, Lupart JL, Marini A. Teaching for transfer: fostering generalization in learning: Routledge; 2013.

[40] Eustache E, Gerbasi M, Fawzi MS, Fils-Aimé J, Severe J, Raviola G (2017). Mental health training for secondary school teachers in Haiti: a mixed methods, prospective, formative research study of feasibility, acceptability, and effectiveness in knowledge acquisition. Global Mental Health..

[41] Shinde S, Weiss HA, Khandeparkar P, Pereira B, Sharma A, Gupta R (2020). A multicomponent secondary school health promotion intervention and adolescent health: an extension of the SEHER cluster randomised controlled trial in Bihar, India. PLoS Med.

[42] Shinde S, Weiss HA, Varghese B, Khandeparkar P, Pereira B, Sharma A (2018). Promoting school climate and health outcomes with the SEHER multi-component secondary school intervention in Bihar, India: a cluster-randomised controlled trial. Lancet.

[43] US Department of Education, editor Office of Special Education and Rehabilitative Services. 39th annual report to Congress on the implementation of the Individuals with Disabilities Education Act; 2017: Office of Special Education Programs Washington, DC.

[44] Landrum TJ, Tankersley M, Kauffman JM (2003). What is special about special education for students with emotional or behavioral disorders?. J Special Education.

[45] Mallison R. Education as Therapy; Suggestions for Work with Neurologically Impaired Children. 1968.

[46] Yoshikawa H, Leyva D, Snow CE, Treviño E, Barata M, Weiland C (2015). Experimental impacts of a teacher professional development program in Chile on preschool classroom quality and child outcomes. Dev Psychol.

[47] Achenbach TM. Manual for the Teacher's Report Form and 1991 profile: Univ Vermont/Department Psychiatry; 1991.

[48] Deb S, Walsh K (2012). Impact of physical, psychological, and sexual violence on social adjustment of school children in India. Sch Psychol Int.

[49] Morrow V, Singh R. Corporal punishment in schools in Andhra Pradesh, India: Children’s and parents’ views: Young Lives; 2014.

