# Supplementary material for: Perceptions, attitudes, and knowledge of teachers serving as mental health lay counselors in a low and middle income country: a mixed methods pragmatic pilot study
Source: Int J Ment Health Syst. 2021 Apr 29;15:40. doi: 10.1186/s13033-021-00453-3 (PMC8082764; doi:10.1186/s13033-021-00453-3)
Supplement: Supplementary file 1 — Additional file 1: Training summative assessment. This is the summative assessment used to assess teacher mental health knowledge PRE, POST, and INT. [file 13033_2021_453_MOESM1_ESM.docx]

Additional File 1

*Training summative assessment*

**Vignette 1**

In school while lining up for morning school assembly first thing in the morning, Rupak asks Sagar to move back; angry, Sagar pushes his friend Rupak until he falls on the ground. Miss Rita notices Sagar pushing Rupak; she then commands Sagar to kneel down during the assembly in front of all the students. She is mad that Sagar pushed his peer again and that she again had to make him kneel in front of all students; he has displayed similar inappropriate behavior all year. She wonders why kneeling in front of all students does not stop him from pushing his peers.

The teacher tries to find the cause for Sagar’s behavior, so she visits Sagar’s house and speaks to his mother asking her about how her day was going with Sagar. Sagar’s mother states that Sagar didn’t wake up on time this morning and was refusing to go to school because he had a math test he was not prepared for. His mother woke him up firmly to get him ready for school and also hit him with a stick because he didn’t study for the test.

The teacher more closely observes Sagar in his math class. She notices that Sagar’s seat is at the back of the class. Miss Rita then shifts Sagar’s seat to the front bench. Over the first few days of sitting on the front bench, Miss Rita notices that Sagar is getting into fewer fights with classmates and is participating more in class.

Miss Rita waits for Sagar to walk home after school and starts conversations by asking questions such as “How are you?”, “How was your day at school?”, and “Did you laugh about anything today?”. After feeling that trust was built between them, Miss Rita then started to find out how he is doing in his math class by asking “Were any subjects difficult today?” Sagar offered that he was struggling with math and further volunteered information on his difficulties, including not liking math, sitting next to distracting peers, and being pressured by his parents to do particularly well in math as they want him to grow up to be an accountant one day. Miss Rita thanks Sagar for sharing his struggles, gives him praise for being brave, and offers to help him with math and with accommodations at school.

1. What trigger immediately precipitates Sagar’s pushing of Rupak? (circle one)

1. Having a math test
2. Waking up late earlier that morning
3. Refusing to go to school
4. Being told to move back in line
5. Being hit with a stick for not studying for the math test

2. What is the root cause of Sagar’s inappropriate behavior the first morning? (circle one)

1. Having a math test
2. Waking up late earlier that morning
3. Refusing to go to school
4. Being told to move back in line
5. Being hit with a stick for not studying for the math test

3. Which one of the following does Sagar obtain by pushing Rupak? (circle one)

1. Positive attention
2. Negative attention
3. Escape
4. Tangible
5. Sensory

4. Fill in the following Cause Analysis chart based on Sagar’s pushing of Rupak the first morning.

| **Antecedent**  *What happens immediately before the behavior?* | **Behavior**   *What did the child do after having the automatic thought?* | **Consequence**  *What happens directly after the behavior?* |
| --- | --- | --- |
|  |  |  |

5. How does the change Miss Rita makes to Sagar’s environment positively affect Sagar’s behavior? (circle all that apply)

1. Sagar interacts more with his peers.
2. Sagar interacts less with his peers.
3. Sagar can better hear the teacher and see the chalkboard.
4. Sagar can leave the classroom more easily.
5. Miss Rita can scold him more easily.

6. How does Miss Rita connect with Sagar? (circle all that apply)

1. Miss Rita spends one on one time with Sagar.
2. Miss Rita shows she is only interested in Sagar’s school performance.
3. Miss Rita publicly punishes Sagar.
4. Miss Rita asks Sagar about his well-being.
5. Miss Rita shows her appreciation for Sagar’s honesty.

7. Cultivating positive behavior is akin to cultivating growth in a tea bush. Below, match the aspects of tea bush growth with the aspects of cultivating positive behavior. Then, match the aspects of cultivating positive behavior with the examples of each in the vignette.

Culture, Relationships

Reward, Nourishment

Limit-setting, Punishment

Pruning

Soil

Water and Sun

*Miss Rita getting to know Sagar and asking him about his point of view*

*Miss Rita giving praise for sharing difficult feelings and offering assistance to help Sagar with his struggles*

*Miss Rita moving Sagar closer to the board but away from his friends*

8. Complete the 4C’s behavior plan below with information from the vignette. Write “none” in the fields for which no information was provided in the vignette.

| **4 C's Behavior Plan** | | |
| --- | --- | --- |
| **Cause** | ***Antecedent theme*** |  |
|  |  |  |
| *What causes the student's behavior to occur?* | ***Consequence theme*** |  |
| **Change** | ***School and/or home*** |  |
|  | ***Organization*** |  |
| *What changes can be made in the environment to help the student succeed?* | ***Classroom learning*** |  |
|  | ***New skills*** |  |
| **Connect** | ***Classroom*** |  |
|  |  |  |
| *How are social connections being made with the student?* | ***One on one*** |  |
| **Cultivate** | ***Rewards*** |  |
|  |  |  |
| *When the student is struggling, what rewards or limits are being used to cultivate the student's positive behavior?* | ***Limits*** |  |

**Vignette 2**

Nishant is an 11-year-old boy who is known to be disruptive and violent. He has hit a boy on the head before to the point of the boy losing consciousness, often hits siblings, disrupts class by yelling at peers or the teacher or dinner by yelling at family, and has occasionally run away from class and from home.

An ABC data sheet was completed for Nishant, and there appeared to be two times where he had the most difficulties, before his science class and after recess. His parents tell the teacher that, at home, he had difficulty with behaving appropriately during dinner. Two scheduled breaks were instituted during the school day for Nishant, one before science and after recess, to give him a chance to regroup during times that have been typically difficult. At home, his parents also instituted 3 five-minute breaks for him, one before, one during, and after dinner. For these “breaks” he a priori chose to either squeeze a squishy ball or being able to walk 10 lengths back and the room he was in. This appeared to improve his outbursts moderately, from every day to 1-2 times per week initially.

Nishant’s teacher then saw him individually once per week; she eventually learned that Nishant was often worried about rejection and praised him effusively when he shared his struggle with her. He admitted that he was often not picked to be part of teams or playgroups at recess. It became clearer to his teacher that Nishant had some difficulty with socializing, including in science where much of the work is in groups. Further, in speaking with his family, the teacher learned that dinner time was the only time in the home that Nishant was tasked with having to discuss his school day, which were often difficult days as they were filled with his worries about rejection. The teacher started coming to Nishant’s recess where she would hold a “special recess group” for Nishant and two other classmates where they would have activities planned including all three boys. The teacher also worked with Nishant’s family to be able to gently and productively discuss Nishant’s school day at dinner without getting Nishant too worked up. Over the course of the year, Nishant’s outbursts were slowly extinguished. He had been able to make better friends with the classmates in his group, was able to discuss his school day at dinner without outbursts, and no longer felt anxious.

9. What is the root cause of Nishant’s inappropriate behavior? (circle one)

1. Talking about his school day
2. Struggling with science subject material
3. Having unstructured time at recess
4. Fear of rejection
5. Spending time with his family

10. How did the breaks change Nishant’s environment and help Nishant decrease his outburst frequency? (circle all that apply)

1. He briefly escaped stressful situations to release tension before returning to the stressful situations more calm.
2. He was able to use self-determined relaxation techniques of squeezing a squishy ball or walking off his energy to help him calm down.
3. He was able to play with peers without a fear of rejection.
4. He was able to become more angry when he had time to himself as he continued to worry about rejection.
5. He was able to complete his science assignments alone.

11. How does the teacher connect with Nishant? (circle all that apply)

1. The teacher spent one on one time with Nishant.
2. The teacher visited Nishant’s home.
3. The teacher set up a playgroup to help Nishant feel more comfortable with peers.
4. The teacher allowed Nishant to escape from science class and not return until the subject was completed.
5. The teacher allowed Nishant to pick activities to complete during his breaks that Nishant knew would help him calm down.

12. How does the teacher cultivate positive behavior in Nishant? (circle all that apply)

1. The teacher set limits with Nishant’s escape; he was able to leave the situation temporarily to calm down while also having to learn how to deal with his worries by having him return to the difficult task.
2. The teacher praised Nishant when he was able to admit to his struggle.
3. The teacher helped create a positive classroom environment for Nishant where he was able to either work alone to avoid social interaction.
4. The teacher helped create a positive environment at home for Nishant where his family could still learn about his school day without fully upsetting Nishant.
5. The teacher created a positive environment at school where, at least once per day, Nishant was included in the peer activity, allowing him to feel more accepted.
