# Supplementary material for: Perceptions, attitudes, and knowledge of teachers serving as mental health lay counselors in a low and middle income country: a mixed methods pragmatic pilot study
Source: Int J Ment Health Syst. 2021 Apr 29;15:40. doi: 10.1186/s13033-021-00453-3 (PMC8082764; doi:10.1186/s13033-021-00453-3)
Supplement: Supplementary file 2 — Additional file 2: Attitudes, knowledge, and perceptions survey. This is the study-specific survey used to assess teacher mental health attitudes, self-perceptions of knowledge, and perceptions of serving as lay counselor. [file 13033_2021_453_MOESM2_ESM.docx]

Additional File 2

*Attitudes, knowledge, and perceptions survey*

Directions: You are being asked to complete this survey to help us evaluate the behavioral health initiative. This information will be used to help us plan future training and consultation sessions. Your participation is voluntary.

To see if your views change over time, we are asking for your name to match your pre-training to your post-training survey and assess the trainings’ impact. After collecting these forms, we will remove your name from both surveys and assign a random code number that will not be linked to your identity. The specific information you provide individually will not be shared with others. Instead, we will report themes across different groups of student support staff.

Name _______________________________________________________

Date________________________________________________________

For the purpose of this survey, we offer the following definition of behavioral health.

**Behavioral health refers to an individual’s emotional wellbeing and to the connection between an individual’s behaviors and the wellbeing of their body, mind, and spirit.**

1. *I have encountered children who struggle with their behavior before.

| Strongly Agree | Moderately Agree | Neither Agree nor Disagree | Moderately Disagree | Strongly Disagree |
| --- | --- | --- | --- | --- |
| 1 | 2 | 3 | 4 | 5 |

2. *I encounter children who struggle with their behavior often.

| Strongly Agree | Moderately Agree | Neither Agree nor Disagree | Moderately Disagree | Strongly Disagree |
| --- | --- | --- | --- | --- |
| 1 | 2 | 3 | 4 | 5 |

3. I have assessed a student for behavioral health concerns before.

| Strongly Agree | Moderately Agree | Neither Agree nor Disagree | Moderately Disagree | Strongly Disagree |
| --- | --- | --- | --- | --- |
| 1 | 2 | 3 | 4 | 5 |

4. I assess students for behavioral health concerns often.

| Strongly Agree | Moderately Agree | Neither Agree nor Disagree | Moderately Disagree | Strongly Disagree |
| --- | --- | --- | --- | --- |
| 1 | 2 | 3 | 4 | 5 |

5. I have interacted one on one with a student with behavioral health concerns to help manage his/her behavior before.

| Strongly Agree | Moderately Agree | Neither Agree nor Disagree | Moderately Disagree | Strongly Disagree |
| --- | --- | --- | --- | --- |
| 1 | 2 | 3 | 4 | 5 |

6. I interact one on one with students with behavioral health concerns to help manage their behavior often.

| Strongly Agree | Moderately Agree | Neither Agree nor Disagree | Moderately Disagree | Strongly Disagree |
| --- | --- | --- | --- | --- |
| 1 | 2 | 3 | 4 | 5 |

7. I have created and implemented a behavior plan to manage the behavior of a student with behavioral health concerns before.

| Strongly Agree | Moderately Agree | Neither Agree nor Disagree | Moderately Disagree | Strongly Disagree |
| --- | --- | --- | --- | --- |
| 1 | 2 | 3 | 4 | 5 |

8. I create and implement behavior plans to manage the behavior of students with behavioral health concerns often.

| Strongly Agree | Moderately Agree | Neither Agree nor Disagree | Moderately Disagree | Strongly Disagree |
| --- | --- | --- | --- | --- |
| 1 | 2 | 3 | 4 | 5 |

9. I have engaged with the family of a student with behavioral health concerns before.

| Strongly Agree | Moderately Agree | Neither Agree nor Disagree | Moderately Disagree | Strongly Disagree |
| --- | --- | --- | --- | --- |
| 1 | 2 | 3 | 4 | 5 |

10. I engage with the families of students with behavioral health concerns often.

| Strongly Agree | Moderately Agree | Neither Agree nor Disagree | Moderately Disagree | Strongly Disagree |
| --- | --- | --- | --- | --- |
| 1 | 2 | 3 | 4 | 5 |

11. I believe that my only job should be to teach students academic subject matter.

| Strongly Agree | Moderately Agree | Neither Agree nor Disagree | Moderately Disagree | Strongly Disagree |
| --- | --- | --- | --- | --- |
| 1 | 2 | 3 | 4 | 5 |

12. *I believe that behavior can be biologically based.

| Strongly Agree | Moderately Agree | Neither Agree nor Disagree | Moderately Disagree | Strongly Disagree |
| --- | --- | --- | --- | --- |
| 1 | 2 | 3 | 4 | 5 |

13. *I believe that the context or environment a person is in can influence the person’s behavior.

| Strongly Agree | Moderately Agree | Neither Agree nor Disagree | Moderately Disagree | Strongly Disagree |
| --- | --- | --- | --- | --- |
| 1 | 2 | 3 | 4 | 5 |

14. *I believe that behavior is a choice one makes.

| Strongly Agree | Moderately Agree | Neither Agree nor Disagree | Moderately Disagree | Strongly Disagree |
| --- | --- | --- | --- | --- |
| 1 | 2 | 3 | 4 | 5 |

15. *I believe that behavior can be changed.

| Strongly Agree | Moderately Agree | Neither Agree nor Disagree | Moderately Disagree | Strongly Disagree |
| --- | --- | --- | --- | --- |
| 1 | 2 | 3 | 4 | 5 |

16. Assessing students for behavioral health concerns is an important part of my job.

| Strongly Agree | Moderately Agree | Neither Agree nor Disagree | Moderately Disagree | Strongly Disagree |
| --- | --- | --- | --- | --- |
| 1 | 2 | 3 | 4 | 5 |

17. Interacting one on one with students with behavioral health concerns to help manage their behavior is an important part of my job.

| Strongly Agree | Moderately Agree | Neither Agree nor Disagree | Moderately Disagree | Strongly Disagree |
| --- | --- | --- | --- | --- |
| 1 | 2 | 3 | 4 | 5 |

18. Creating and implementing behavior plans to manage the behavior of students with behavioral concerns is an important part of my job.

| Strongly Agree | Moderately Agree | Neither Agree nor Disagree | Moderately Disagree | Strongly Disagree |
| --- | --- | --- | --- | --- |
| 1 | 2 | 3 | 4 | 5 |

19. Engaging with families of students with behavioral health concerns is an important part of my job.

| Strongly Agree | Moderately Agree | Neither Agree nor Disagree | Moderately Disagree | Strongly Disagree |
| --- | --- | --- | --- | --- |
| 1 | 2 | 3 | 4 | 5 |

20. Having to deal with my students’ behavioral health concerns is burdensome to me.

| Strongly Agree | Moderately Agree | Neither Agree nor Disagree | Moderately Disagree | Strongly Disagree |
| --- | --- | --- | --- | --- |
| 1 | 2 | 3 | 4 | 5 |

21. – 24. I am knowledgeable about the following behavioral health concerns:

| Behavior Concern | Strongly Agree | Moderately Agree | Neither Agree nor Disagree | Moderately Disagree | Strongly Disagree |
| --- | --- | --- | --- | --- | --- |
| Disruptive behavior | 1 | 2 | 3 | 4 | 5 |
| Aggression | 1 | 2 | 3 | 4 | 5 |
| Suicidal thoughts | 1 | 2 | 3 | 4 | 5 |
| Withdrawn behavior | 1 | 2 | 3 | 4 | 5 |

25. – 28. I am effective in assessing students with the following behavioral health concerns:

| Behavior Concern | Strongly Agree | Moderately Agree | Neither Agree nor Disagree | Moderately Disagree | Strongly Disagree |
| --- | --- | --- | --- | --- | --- |
| Disruptive behavior | 1 | 2 | 3 | 4 | 5 |
| Aggression | 1 | 2 | 3 | 4 | 5 |
| Suicidal thoughts | 1 | 2 | 3 | 4 | 5 |
| Withdrawn behavior | 1 | 2 | 3 | 4 | 5 |

29. Assessing students with behavioral health concerns is up to me.

| Strongly Agree | Moderately Agree | Neither Agree nor Disagree | Moderately Disagree | Strongly Disagree |
| --- | --- | --- | --- | --- |
| 1 | 2 | 3 | 4 | 5 |

30. My lack of knowledge about behavioral health issues keeps me from assessing students with behavioral health concerns.

| Strongly Agree | Moderately Agree | Neither Agree nor Disagree | Moderately Disagree | Strongly Disagree |
| --- | --- | --- | --- | --- |
| 1 | 2 | 3 | 4 | 5 |

31. – 34. I am effective in interacting one on one with students with the following behavioral health concerns:

| Behavior Concern | Strongly Agree | Moderately Agree | Neither Agree nor Disagree | Moderately Disagree | Strongly Disagree |
| --- | --- | --- | --- | --- | --- |
| Disruptive behavior | 1 | 2 | 3 | 4 | 5 |
| Aggression | 1 | 2 | 3 | 4 | 5 |
| Suicidal thoughts | 1 | 2 | 3 | 4 | 5 |
| Withdrawn behavior | 1 | 2 | 3 | 4 | 5 |

35. Interacting one on one with students is an effective way to manage students with behavioral health concerns.

| Strongly Agree | Moderately Agree | Neither Agree nor Disagree | Moderately Disagree | Strongly Disagree |
| --- | --- | --- | --- | --- |
| 1 | 2 | 3 | 4 | 5 |

36. My lack of knowledge about one on one interactions with students with behavioral health concerns keeps me from interacting one on one with students with behavioral health concerns.

| Strongly Agree | Moderately Agree | Neither Agree nor Disagree | Moderately Disagree | Strongly Disagree |
| --- | --- | --- | --- | --- |
| 1 | 2 | 3 | 4 | 5 |

37. I am effective in creating and implementing behavior plans for students with the following behavioral health concerns:

| Behavior Concern | Strongly Agree | Moderately Agree | Neither Agree nor Disagree | Moderately Disagree | Strongly Disagree |
| --- | --- | --- | --- | --- | --- |
| Disruptive behavior | 1 | 2 | 3 | 4 | 5 |
| Aggression | 1 | 2 | 3 | 4 | 5 |
| Suicidal thoughts | 1 | 2 | 3 | 4 | 5 |
| Withdrawn behavior | 1 | 2 | 3 | 4 | 5 |

38. Creating and implementing behavior plans is an effective way to manage students with behavioral health concerns.

| Strongly Agree | Moderately Agree | Neither Agree nor Disagree | Moderately Disagree | Strongly Disagree |
| --- | --- | --- | --- | --- |
| 1 | 2 | 3 | 4 | 5 |

39. My lack of knowledge about behavior plans keeps me from creating and implementing behavior plans for students with behavioral health concerns.

| Strongly Agree | Moderately Agree | Neither Agree nor Disagree | Moderately Disagree | Strongly Disagree |
| --- | --- | --- | --- | --- |
| 1 | 2 | 3 | 4 | 5 |

40. – 43. I am effective in engaging with families of students with the following behavioral health concerns:

| Behavior Concern | Strongly Agree | Moderately Agree | Neither Agree nor Disagree | Moderately Disagree | Strongly Disagree |
| --- | --- | --- | --- | --- | --- |
| Disruptive behavior | 1 | 2 | 3 | 4 | 5 |
| Aggression | 1 | 2 | 3 | 4 | 5 |
| Suicidal thoughts | 1 | 2 | 3 | 4 | 5 |
| Withdrawn behavior | 1 | 2 | 3 | 4 | 5 |

44. Engaging with families of students with behavioral health concerns is an effective way to manage students with behavioral health concerns.

| Strongly Agree | Moderately Agree | Neither Agree nor Disagree | Moderately Disagree | Strongly Disagree |
| --- | --- | --- | --- | --- |
| 1 | 2 | 3 | 4 | 5 |

45. My lack of knowledge about engaging with families of students with behavioral concerns keeps me from engaging with families of students with behavioral health concerns.

| Strongly Agree | Moderately Agree | Neither Agree nor Disagree | Moderately Disagree | Strongly Disagree |
| --- | --- | --- | --- | --- |
| 1 | 2 | 3 | 4 | 5 |

46. I intend to assess my students for behavioral health concerns.

| Strongly Agree | Moderately Agree | Neither Agree nor Disagree | Moderately Disagree | Strongly Disagree |
| --- | --- | --- | --- | --- |
| 1 | 2 | 3 | 4 | 5 |

47. I intend to interact one on one with students with behavioral health concerns.

| Strongly Agree | Moderately Agree | Neither Agree nor Disagree | Moderately Disagree | Strongly Disagree |
| --- | --- | --- | --- | --- |
| 1 | 2 | 3 | 4 | 5 |

48. I intend on creating and implementing behavior plans for students with mental health concerns.

| Strongly Agree | Moderately Agree | Neither Agree nor Disagree | Moderately Disagree | Strongly Disagree |
| --- | --- | --- | --- | --- |
| 1 | 2 | 3 | 4 | 5 |

49. I intend on engaging the families of students with behavioral health concerns.

| Strongly Agree | Moderately Agree | Neither Agree nor Disagree | Moderately Disagree | Strongly Disagree |
| --- | --- | --- | --- | --- |
| 1 | 2 | 3 | 4 | 5 |

What has been the most helpful aspect of the behavioral health trainings for you?

What would make the behavioral trainings more helpful to you?

Any other feedback you would like to share?
